# Discordance between germline genetic findings and abnormal tumor immunohistochemistry staining of mismatch repair proteins in individuals with suspected Lynch syndrome

**DOI:** 10.3389/fonc.2023.1069467

**Published:** 2023-01-30

**Authors:** Shujuan Pan, Hannah Cox, Jamie Willmott, Erin Mundt, Heidi Gorringe, Michelle Landon, Karla R. Bowles, Bradford Coffee, Benjamin B. Roa, Debora Mancini-DiNardo

**Affiliations:** Myriad Genetic Laboratories, Inc., Salt Lake City, UT, United States

**Keywords:** hereditary cancer syndrome, clinical genetic testing, cancer diagnosis, universal tumor screening, IHC – immunohistochemistry

## Abstract

**Background and Aims:**

Tumor immunohistochemical staining (IHC) of DNA mismatch repair (MMR) proteins is often used to guide germline genetic testing and variant classification for patients with suspected Lynch syndrome. This analysis examined the spectrum of germline findings in a cohort of individuals showing abnormal tumor IHC.

**Methods:**

We assessed individuals with reported abnormal IHC findings and referred for testing with a six-gene syndrome-specific panel (n=703). Pathogenic variants (PVs) and variants of uncertain significance (VUS) in MMR genes were designated expected/unexpected relative to IHC results.

**Results:**

The PV positive rate was 23.2% (163/703; 95% confidence interval [CI], 20.1%-26.5%); 8.0% (13/163; 95% CI, 4.3%-13.3%) of PV carriers had a PV in an unexpected MMR gene. Overall, 121 individuals carried VUS in MMR genes expected to be mutated based on IHC results. Based on independent evidence, in 47.1% (57/121; 95% CI, 38.0%-56.4%) of these individuals the VUSs were later reclassified as benign and in 14.0% (17/121; 95% CI, 8.4%-21.5%) of these individuals the VUSs were reclassified as pathogenic.

**Conclusions:**

Among patients with abnormal IHC findings, IHC-guided single-gene genetic testing may miss 8% of individuals with Lynch syndrome. In addition, in patients with VUS identified in MMR genes predicted to be mutated by IHC, extreme caution must be taken when the IHC results are considered in variant classification.

## Introduction

Lynch syndrome is caused by an inherited germline pathogenic variant (PV) in one or more of the DNA mismatch repair (MMR) genes *MLH1*, *MSH2*, *MSH6*, and *PMS2* (1, 2). Approximately 3% of colorectal cancer (CRC) cases result from Lynch syndrome, making it the most common heritable CRC syndrome. Lynch syndrome also is associated with endometrial, ovarian, gastric/small bowel, urothelial, central nervous system, pancreatic, and prostate cancers (3). Clinical management for patients with a Lynch syndrome-related cancer involves heightened secondary cancer surveillance and can include risk-reducing surgeries – measures that have been shown to reduce morbidity and mortality (4–6). Therefore, it is essential to distinguish between Lynch syndrome and sporadic disease in patients diagnosed with cancer.

One first-line approach to differentiate between Lynch syndrome and sporadic cancer is to use immunohistochemical (IHC) staining to assess MLH1, MSH2, MSH6, and PMS2 protein expression in tumor tissue from biopsy or surgical resection. The National Comprehensive Cancer Network (NCCN), the American Society for Clinical Oncology, and others recommend universal IHC screening of new CRC and endometrial cancer cases (3, 7). Abnormal tumor MMR protein expression by IHC suggests a deficiency in the corresponding gene(s) and compromised MMR. NCCN recommends referral for further genetic testing for patients with abnormal IHC findings (3, 8–11).

Reflex genetic testing after an abnormal tumor IHC result can follow numerous paths. For example, MLH1 protein expression can be disrupted either by a germline pathogenic variant (PV) in *MLH1*, DNA promoter hypermethylation that silences the gene, or by double somatic mutations. Historically, if MLH1 is absent on IHC for CRC tumors, subsequent testing can take several directions: (1) germline *MLH1* testing; (2) tumor *MLH1* methylation testing; (3) tumor testing for the BRAF p.V600E PV based on its association with *MLH1* methylation status (3, 12). In recent years, tumor testing of the MMR genes to detect somatic mutations in MMR genes has also been recommended (13). Nevertheless, only when gene-specific testing fails to identify a mutation will germline testing of additional MMR genes and/or other genes associated with hereditary cancer syndromes typically be recommended (3). This stepwise approach has proven complex, confusing, and time-consuming. While NCCN guidelines recommend that an individual with expertise in genetics be involved in the diagnostic process (3), surveyed gastroenterologists reported that it is often unclear which specialist would be responsible for selecting and ordering the test (14). Each separate test adds time to the patient’s diagnostic journey and increases the risk of loss to follow-up, which can delay or prevent risk-reducing surgical procedures.

Another relevant concern is the sensitivity of MMR IHC, with a 5-10% false negative rate (3, 15). Staining quality can vary depending on the tumor microenvironment and tissue fixation conditions, leading to ambiguity, misinterpretation of results, and misinformed gene selection for testing (16). In addition, some individuals who have abnormal IHC are found to carry germline PVs in MMR genes not predicted by the IHC result (17–19) or in non-MMR genes associated with other cancer syndromes (20, 21), which would have been missed using gene-specific genetics testing guided by IHC results.

In addition to its use as a screening tool, IHC results may be employed as supportive evidence in determining the pathogenicity of variants identified in genes predicted by IHC to be mutated (22). For instance, the variant classification criteria used by the International Society for Gastrointestinal Hereditary Tumors (InSiGHT) indicate that: when the presence of a variant of uncertain significance (VUS) coincides with the absence of the corresponding MMR protein on IHC in two or more patients, it is deemed supporting evidence for class 5 (pathogenic) or class 4 (likely pathogenic); conversely, inconsistent IHC results observed in three or more tumors were considered as supportive evidence for class 2 (likely benign) or class 1 (benign) (23). This application is concerning given the low predictive value of IHC staining for Lynch syndrome (24, 25). Although IHC results generally are not used as stand-alone evidence for variant classification, there exists potential for an incorrect determination (22).

The frequency of discordance between IHC results and germline genetic findings across various tumor types has not been evaluated systematically in the clinical laboratory, meaning that it is unclear how many patients might be affected clinically by incomplete or misleading IHC results. The current analysis aimed to address this knowledge gap by evaluating germline genetic findings from multi-gene panel testing of individuals with abnormal IHC results in Lynch-associated tumor types. The objectives were to determine (1) the extent of PVs in genes not predicted by IHC, and (2) the possibility of misclassifying variants based on IHC findings.

## Materials and methods

### Patient population

The analysis included individuals who underwent clinical genetic testing that included the MMR genes (*MLH1*, *MSH2*, *MSH6*, *PMS2*) from May 2011 through April 2018. Individuals were included if they reported a personal history of cancer (e.g., colorectal, endometrial, ovarian) and/or colorectal polyps and an abnormal MMR IHC test result in a tumor sample type indicated for Lynch syndrome IHC testing (i.e., CRC or endometrial cancer). To eliminate pre-existing mutation bias and ensure the mutation status of all MMR genes were obtained, only patients whose genetic testing included all four genes were assessed. Therefore, the following criteria were not part of our data query: individuals who were tested for a subset of the MMR genes, for ancestry-specific founder mutations, or for a known familial mutation. Testing was performed by Myriad Genetic Laboratories, Inc. (Salt Lake City, UT), a national Clinical Laboratory Improvement Amendments- and College of American Pathology-certified facility. All individuals provided consent for clinical genetic testing, and test data were de-identified and aggregated for analysis. As a retrospective study performed on de-identified samples, this analytical validation was not subject to any additional review (HHS regulation 45 CFR 46 per section § 46.101). Clinical information, including personal history of cancer and the IHC tumor test result, was obtained from the test request form completed by the healthcare provider.

### Genetic testing and variant classification

Genomic DNA was extracted from each patient’s blood sample (QIAsymphony; Qiagen, Venlo, The Netherlands), and subjected to genetic testing using a six-gene cancer panel designed for individuals with suspected Lynch syndrome or *MUTYH*-associated polyposis. The panel included *MLH1*, *MSH2*, *MSH6*, *PMS2*, *EPCAM*, and *MUTYH*. Testing included sequencing and large rearrangement analysis of all genes except *EPCAM* (large rearrangement analysis only).

For sequencing analysis, exonic regions and adjacent -20/+10 intron regions of each gene were amplified by Polymerase Chain Reaction (PCR) and sequenced in forward and reverse directions. For *PMS2* exons 11-15 that have high homology to pseudogenes, a long-range PCR was first performed, and the regions of interest were amplified by nested PCR followed by sequencing.

For large rearrangement analysis of *MLH2*, *MSH2*, *MSH6*, *EPCAM* and *MUTYH*, a clinically validated high-density oligonucleotide microarray was used as the primary methodology (26) and multiplex ligation-dependent probe amplification (MLPA) (MRC-Holland, Amsterdam, The Netherlands) was used as confirmatory approach. For large rearrangements in *PMS2*, MLPA was used as the primary methodology. For any copy number changes revealed by MLPA in the pseudogene region of *PMS2*, long-range PCR was also performed to determine whether the large rearrangement was in *PMS2* or the pseudogene.

Variant classification was consistent with guidelines from the American College of Medical Genetics and Genomics, as previously described (27, 28). Variants with a laboratory classification of pathogenic or likely pathogenic were considered PVs. Variants with a laboratory classification of benign or likely benign were considered benign (i.e., clinically insignificant). Variants for which clinical significance could not be determined were classified as VUS.

### Analysis

Genetic test results were considered “expected” if a germline PV was detected in an MMR gene consistent with the gene-specific testing strategy recommended by NCCN guidelines for the IHC test result (**Supplemental Table 1**) (3). For example, detection of a germline *MLH1* or *PMS2* mutation was considered “expected” in an individual who showed loss of MLH1 and/or PMS2 on IHC; however, a germline *MSH2* mutation in this individual would be considered “unexpected”. The analysis also partitioned results based on “typical” and “atypical” MMR IHC patterns. In general, typical IHC patterns were those that involved only one of the two characteristic MMR heterodimer pairs, MSH2/MSH6 or MLH1/PMS2, and listed in the NCCN guidelines. Atypical patterns involved MMR proteins from both MMR heterodimer pairs. For proportions, an exact 95% confidence interval (CI) was calculated.

## Results

### Analysis group characteristics

A total of 703 individuals were included in this analysis. **Table 1** shows clinical and demographic characteristics according to the genetic test performed. Overall, CRC and endometrial were the most common cancers for these individuals, with CRC diagnosed in 76% and endometrial cancer in 23.5% of these individuals. Many individuals were diagnosed with two or more cancer types (e.g. CRC and endometrial cancer, endometrial cancer and ovarian cancer etc.) or one cancer type plus colorectal polyps. The majority of individuals were female (61.5%, N=432) and the median age at genetic testing was 59.2 years.

**Table 1:** Characteristics of the testing population.

	Patient characteristics (N=703)
Age at testing (years)	
Mean	59.4
Median	59.2
Range	15.3-91.5
Gender [n (%)]	
Male	269 (38.3)
Female	432 (61.5)
Not specified	2 (0.3)
Personal cancer history^a^, n (%)	
Colorectal	534 (76.0)
Endometrial	165 (23.5)
Ovarian	7 (1.0)
Other	119 (16.9)
Colorectal polyps	96 (13.7)
Not specified^c^	1 (0.1)
Age at diagnosis (years)^b^, n (%)	
≤40	81 (12.1)
41-50	136 (20.4)
51-60	163 (24.4)
61-70	142 (21.3)
>70	130 (19.5)
Not specified	15 (2.2)

a Individuals with multiple cancer diagnoses are included in each appropriate row.

b Earliest age at cancer diagnosis; includes only individuals with colorectal, endometrial, or ovarian cancer.

c Tumor type is not specified on the test request form.

Fifteen distinct abnormal IHC patterns were reported. This included six IHC patterns categorized as typical, involving proteins from only one MMR heterodimer pair, and nine categorized as atypical, involving proteins from both pairs (**Table 2**). In total, 84.8% (N=596/703) of reported IHC patterns were typical, with the most common being a lack of MLH1/PMS2 expression. Among the 15.2% (N=107/703) atypical patterns, the most common involved disrupted expression of all four MMR proteins. Since atypical IHC patterns were rare, they were combined for subsequent analyses.

**Table 2:** Immunohistochemistry patterns for MMR proteins among tested individuals.

	n (%)
Typical IHC patterns^a^	596 (84.8)
MLH1/PMS2	265 (37.7)
MSH2/MSH6	103 (14.7)
MSH6	92 (13.1)
PMS2	72 (10.2)
MLH1	40 (5.7)
MSH2	24 (3.4)
Atypical IHC patterns^b^	107 (15.2)
MLH1/MSH2/MSH6/PMS2	34 (4.8)
MLH1/MSH6/PMS2	17 (2.4)
MSH6/PMS2	23 (3.3)
MLH1/MSH2	8 (1.1)
MLH1/MSH2/MSH6	10 (1.4)
MSH2/MSH6/PMS2	7 (1.0)
MLH1/MSH2/PMS2	4 (0.6)
MLH1/MSH6	2 (0.3)
MSH2/PMS2	2 (0.3)
**Total**	**703**

a Typical patterns involved only one of the two MMR heterodimers (MSH2/MSH6 or MLH1/PMS2).

b Atypical patterns involved two or three MMR proteins that were from both MMR heterodimer pairs.

IHC, immunohistochemistry; MMR, mismatch repair.

### Germline PV identification

Among all the individuals included in this study, 23.2% (N=163/703; 95% CI, 20.1%-26.5%) carried germline PVs in MMR genes (**Table 3**). Expected PVs in MMR genes were seen in 21.3% individuals (N=150/703). Among the 163 PV carriers, 8.0% (N=13/163; 95% CI, 4.3%-13.3%) carried PVs in unexpected MMR genes. No individual carried more than one PV, and no *EPCAM* PVs were identified in this cohort. Monoallelic PVs in *MUTYH* were identified in 6 individuals (**Supplemental Table 2**). These were excluded from the analysis because only biallelic *MUTYH* PVs are considered as high risk for CRC (29, 30).

**Table 3:** Expected or unexpected germline pathogenic variants (PVs) identified among individuals tested with the six-gene panel, distributed by IHC pattern and type of variant.

Tested Individuals				
IHC pattern	Total	With PV N (%)	Individuals with PV in expected^a^ MMR gene N (% of 703)	Individuals with PV in unexpected^a^ MMR gene N (% of 703)
Typical				
MLH1/PMS2	265	31 (11.7)	28 (10.6%)	3 (1.1%)
MSH2/MSH6	103	33 (32.0)	31 (30.1%)	2 (1.9%)
MSH6	92	42 (45.7)	42 (45.7%)	0 (0%)
PMS2	72	23 (31.9)	23 (31.9%)	0 (0%)
MLH1	40	5 (12.5)	3 (7.5%)	2 (5%)
MSH2	24	10 (41.7)	5 (20.8%)	5 (20.8%)
Atypical^b^	107	19 (17.8)	18 (16.8%)	1 (0.9%)
**Total**	703	163 (23.2)	150 (21.3%)	13 (1.8%)

a Based on IHC result.

b Atypical IHC patterns are listed in **Table 2**.

**Table 3** shows the distribution of IHC patterns among individuals found to have expected or unexpected MMR-gene PVs. It appears that PVs in expected MMR genes occurred most frequently in individuals showing isolated loss of MSH6 on IHC (45.7%; N=42/92) and least frequently among those showing isolated loss of MLH1 (7.5%; N=3/40). The most frequent unexpected MMR findings were observed in individuals who had isolated loss of MSH2 on IHC (20.8%; N=5/24). No unexpected MMR mutations were found in individuals with loss of MSH6 or PMS2 on IHC.

**Table 4** lists the 13 individuals with unexpected germline PVs. The most common unexpected finding was germline PVs in *MSH6* in individuals with isolated loss of MSH2 on IHC, which was seen in 4 out of 5 individuals.

**Table 4:** Unexpected germline mismatch repair gene pathogenic variants found in individuals with different immunohistochemistry patterns.

IHC pattern	Germline PV				
	*MLH1*	*MSH2*	*MSH6*	*PMS2*	Total
MLH1	0	2	0	0	2
MLH1/MSH2/PMS2	0	0	1	0	1
MLH1/PMS2	0	2	1	0	3
MSH2	0	0	4	1	5
MSH2/MSH6	1	0	0	1	2
Total	1	4	6	2	13

Monoallelic MUTYH PVs are provided in **Supplemental Table 2**.

### Germline VUS in MMR genes

199 MMR-gene VUSs were identified in nearly a quarter of the cohort (24.0%; N=169/703; 95% CI, 20.9%-27.4%) and some patients harbored more than one VUS. In 121 patients, 132 of these VUSs occurred in MMR genes that are expected to be mutated based on the IHC test result (17.2%; N=121/703; 95% CI,14.5%-20.2%).

For these 132 VUSs, there was a lack of sufficient evidence to support pathogenic or benign classifications at the time of variant identification (IHC was not considered as evidence for classification). During the timeline of this data query, May 2011 through April 2018, 44.7% (N=59/132; 95% CI, 36.0%-53.6%) of these observed VUSs were re-classified to likely benign or benign using evidence independent of IHC results, affecting 47.1% (N=57/121) individuals. By contrast, only 12.9% (N=17/132) of these VUSs were re-classified to PVs, affecting 14.0% (17/121) individuals **(** **Table 5** **)**. The percentages of re-classified VUSs varied between different IHC patterns. For example, in individuals with isolated loss of PMS2 on IHC, 55.6% of *PMS2* VUSs were downgraded to benign/likely benign variants. However, only 16.7% *MLH1* VUSs were downgraded to benign/likely benign in patients with isolated loss of MLH1. On the other hand, only 3.6% of *MSH6* VUSs were upgraded to PVs in patients with loss of MSH6 in IHC; however, 50% of *MSH2* VUSs found in individuals with loss of MSH2 were determined to be pathogenic (**Table 5**).

**Table 5:** Germline MMR gene VUSs that were observed in at least one individual with consistent IHC according to IHC pattern.

	Observed variants			Individuals carrying the variants		
IHC pattern	Total	Downgraded to Benign/Likely Benign	Upgraded to Pathogenic/Likely Pathogenic	Total	Downgraded to Benign/Likely Benign	Upgraded to Pathogenic/Likely Pathogenic
Typical						
MLH1; PMS2	42	22 (52.4%)	5 (11.9%)	40	22 (55.0%)	5 (12.5%)
MSH2; MSH6	21	9 (42.9%)	4 (19.0%)	20	8 (40.0%)	4 (20.0%)
MSH6	28	8 (28.6%)	1 (3.6%)	24	8 (23.3%)	1 (4.2%)
PMS2	18	10 (55.6%)	4 (22.2%)	15	9 (60.0%)	4 (26.7%)
MLH1	6	1 (16.7%)	1 (16.7%)	5	1 (20.0%)	1 (20.0%)
MSH2	2	1 (50.0%)	1 (50.0%)	2	1 (50.0%)	1 (50.0%)
Atypical^a^	15	8 (44.4%)	1 (6.7%)	15	8 (44.4%)	1 (6.7%)
**Total**	132	59 (44.7%)	17 (12.9%)	121	57 (47.1%)	17 (14.0%)

a Atypical IHC patterns are listed in **Table 2**.

These 132 VUSs represented 103 unique variants, 35 of which were downgraded and 14 of which were upgraded (**Table 6**). InSiGHT guidelines indicate that IHC can be considered as evidence in variant classification if a VUS is observed in at least two patients when the gene is expected to be mutated based on IHC results (23). To assess the potential impact of IHC results in variant classification consistent with InSiGHT criteria, we evaluated the variants in genes expected to be mutated based on IHC in at least two patients. Of the 103 unique VUS, 17 were observed in at least 2 patients. Among these 17 variants that were classified as VUSs at the time of identification, 70.6% (N=12/17; 95% CI, 44.0%-89.7%) were downgraded to benign/likely benign and 17.6% (N=3/17; 95% CI, 3.8%-43.4%) were upgraded to pathogenic/likely pathogenic. The downgrade affected 77.8% (N=35/45; 95% CI, 62.9%-88.8%) of patients, and the upgrade affected 13.3% (N=6/45; 95% CI, 5.1%-26.8%) of patients (**Table 6**).

**Table 6:** Germline MMR gene VUS that were reclassified and patients affected by reclassification.

	Consistent IHC in ≥ 1 Patient	Consistent IHC in ≥ 2 Patient
Unique variants		
Total	103	17
Downgraded to Benign/Likely Benign, N (%)	35 (34.0%)	12 (70.6%)
Upgraded to Pathogenic/Likely Pathogenic, N (%)	14 (13.6%)	3 (17.6%)
Total patients with expected MMR variants (includes multiple observations of the same variant)		
Total	121	45
Downgraded to Benign/Likely Benign, N (%)	57 (47.1%)	35 (77.8%)
Upgraded to Pathogenic/Likely Pathogenic, N (%)	17 (14.0%)	6 (13.3%)

The evidence used for downgrading a VUS to benign/likely benign included an in-house cancer history weighting algorithm (Pheno) (31), in-trans observation with a PV in patients with no features of constitutional mismatch repair deficiency (CMMRD) syndrome (phase), functional RNA studies (splicing), updated population frequency estimate (population), and in-house algorithm for using multiple co-occurrence for evidence of pathogenicity called MCO (28, 31). **Table 7** lists the basis for downgrade for these 35 variants. The most frequently used evidence was Pheno, accounting for downgrade of 17 variants. Downgrading based on phase (i.e., in-trans findings) in patients without clinical features of CMMRD was used for 13 variants. Seven variants were downgraded using population frequency.

**Table 7:** 35 variants downgraded from VUS to Benign/Likely benign.

Gene	Variant	Current classification	Evidence used for downgrade
*MLH1*	c.977T>C (p.Val326Ala)	PM	Pheno and MCO
*MSH6*	c.3203G>A (p.Arg1068Gln)	Likely benign	MCO
*MLH1*	c.1321G>A (p.Ala441Thr)	PM	MCO and Phase
*MSH2*	c.1465G>A (p.Glu489Lys)	PM	MCO and Pheno
*MSH6*	c.3173-18T>A	PM	MCO and Pheno
*MSH6*	c.-18G>T	PM	MCO and Phase
*PMS2*	c.2149G>A (p.Val717Met)	Likely benign	Phase
*PMS2*	c.2356C>A (p.Leu786Met)	Likely benign	Phase
*MLH1*	c.1732-19T>A	PM	Phase
*MLH1*	c.1897-17C>G	PM	Phase, Pheno
*MSH2*	c.815C>T (p.Ala272Val)	PM	Phase, Pheno
*MSH2*	c.1168C>T (p.Leu390Phe)	PM	Phase
*MLH1*	c.1360G>C (p.Gly454Arg)	PM	Pheno
*MSH6*	c.1474A>G (p.Met492Val)	Likely benign	Pheno
*MSH2*	c.877A>G (p.Thr293Ala)	Likely benign	Pheno
*MLH1*	c.2066A>G (p.Gln689Arg)	Likely benign	Pheno
*MLH1*	c.1268G>A (p.Arg423Lys)	Likely benign	Pheno
*MSH6*	c.1844G>C (p.Cys615Ser)	Likely benign	Pheno
*MSH2*	c.160G>T (p.Ala54Ser)	Likely benign	Pheno
*MLH1*	c.1667+4A>G	Likely benign	Pheno
*MSH2*	c.1600C>T (p.Arg534Cys)	Likely benign	Pheno
*PMS2*	c.251-20T>G	Likely benign	Population
*PMS2*	c.52A>G (p.Ile18Val)	PM	Population
*MLH1*	c.307-19A>G	PM	Splicing
*MLH1*	c.1963A>G (p.Ile655Val)	PM	MCO,Phase
*MSH6*	c.3160A>T (p.Ile1054Phe)	PM	Population
*PMS2*	c.1437C>G (p.His479Gln)	PM	Population
*PMS2*	c.1711C>A (p.Leu571Ile)	PM	Population
*MSH2*	c.380A>G (p.Asn127Ser)	PM	Phase,Pheno
*MSH2*	c.1321A>C (p.Thr441Pro)	PM	Phase,Pheno
*MSH6*	c.2633T>C (p.Val878Ala)	PM	Phase
*PMS2*	c.1609G>A (p.Glu537Lys)	PM	Population
*MSH6*	c.3488A>T (p.Glu1163Val)	PM	Population
*MSH2*	c.1387-8G>T	PM	Phase
*MSH2*	c.1277-8T>C	PM	Pheno

## Discussion

In this study, we analyzed germline findings in 703 individuals with Lynch syndrome-associated cancer types and abnormal IHC findings to evaluate the concordance of IHC with the germline findings. To our knowledge, this is the first study to show the prevalence and spectrum of germline MMR gene mutations detected in a heterogeneous population with abnormal IHC that are referred to a commercial molecular diagnostic laboratory.

Within the entire cohort, only 21.3% of the individuals carried PVs in MMR genes predicted by IHC. This is lower than the previously reported germline PV positive rates in CRC patients with combined IHC and somatic BRAF testing (13). Somatic BRAF mutation p.V600E is present in 69% of methylation cases (32), which can contribute to the absence of MLH1/PMS2 on IHC. This study is solely based on the IHC results provided by the health care provider on the test requisition forms without any information on the somatic *BRAF* mutation status, therefore many of the cases with MLH1/PMS2 missing on IHC may be resulting from a somatic *BRAF* mutation. This might contribute to the lower PV positive rate in our cohort as we did not have information on somatic *BRAF* mutation status.

Overall, 8% of PV carriers identified by our panel testing carry a PV in an MMR gene not predicted by IHC results. This is particularly prevalent in individuals with isolated loss of MSH2 by IHC, where a single gene testing strategy would lead to *MSH2* sequencing. Of the 10 individuals with loss of MSH2 by IHC and PV positive in this cohort, 5 harbored PVs in genes other than *MSH2*. These findings suggests that IHC-guided single gene testing can extend the patient’s diagnostic journey, potentially miss a Lynch syndrome diagnosis, and delay appropriate medical management. Therefore, MMR gene panel tests should be offered to all patients with abnormal IHC to prevent missing a Lynch syndrome diagnosis.

Our data showed that over 17% of patients with abnormal IHC had a VUS initially identified in the MMR gene predicted to be mutated by IHC. More importantly, nearly 1/3 of these variants were observed in more than one individual with concordant IHC results, which would be considered supporting evidence for a class 5 (pathogenic) or a class 4 (likely pathogenic) classification according to InSiGHT guidelines (23). However, in nearly half of these patients, the VUSs identified were downgraded to benign variants. If IHC results had been used as evidence for pathogenicity, these variants might have been classified in error as likely pathogenic, potentially leading to unnecessary overtreatment in the form of intensified screening and risk-reducing surgeries. These findings warrant great caution for the use of IHC results as evidence of pathogenicity for variants in MMR genes.

We observed 15 different IHC patterns in our patient cohort, 9 of which we considered “atypical” because proteins from both MLH1/PMS2 and MSH2/MSH6 heterodimers were affected. It has been reported that the IHC-null phenotype, in which all four MMR proteins are absent, can be caused by a combination of *MLH1* promoter methylation and double somatic mutation in *MSH2*(33). Double somatic mutations have been demonstrated as an important mechanism affecting MMR protein expression (13, 34, 35). We suspect that many of these atypical IHC patterns in our cohort are caused by this mechanism, affecting both heterodimers; or in certain cases by a combination of a germline mutation affecting one heterodimer and double somatic mutation affecting the other heterodimer. Some of these IHC patterns, such as isolated loss of MLH1, may represent certain artifacts due to antibody reactivity (36). Nevertheless, the findings of PVs in these cases underscores the necessity of testing all MMR genes for patients suspected for Lynch syndrome, irrespective of the IHC results.

One limitation of the analysis was the assumption that the IHC results reported on the test request form were accurate and that results were based on a staining method that included all four MMR proteins. In practice, to reduce costs, laboratories often begin with two-protein staining for MSH6 and PMS2, with reflex to MSH2 and/or MLH1 if a defect is detected. The rationale for two-protein staining stems from the fact that the stabilities of MSH6 and PMS2 depend largely on their dimerization with MSH2 and MLH1, respectively. However, it has been shown that the two-staining method can miss a small number of Lynch syndrome patients who have solitary loss of MSH2 (37). Based on this observation, patients with intact PMS2 or MSH6, but isolated loss of MLH1 or MSH2 may have been inadvertently excluded from this patient cohort. However, these findings are considered rare, and since they are not included in the concordance calculation, we do not anticipate these patients to greatly impact our conclusion. Another limitation of our study is the lack of information of the *MLH1* promoter methylation status. Promoter methylation assays often are conducted for individuals showing loss of MLH1 and/or PMS2 patterns, and only upon testing negative for promoter methylation would these individuals be referred for germline genetic testing (17, 38, 39). However, *MLH1* promoter methylation status was unknown for individuals in this study since methylation assay information was not captured on the laboratory’s test request form. Therefore, the low PV-positive rates correlating with loss of MLH1 or concurrent loss of MLH1/PMS2 on IHC might reflect *MLH1* promoter methylation in some individuals.

In conclusion, the overall germline PV positive rate of abnormal IHC in a population of patients with Lynch-associated cancer types who were referred to a clinical molecular diagnostic lab is approximately 20%, and nearly 2% of these individuals carried a germline PV in an MMR gene that was not consistent with the IHC result. In addition, VUS findings in genes that appear consistent with IHC findings were often downgraded to benign based on independent evidence. These findings raise two clinical issues. First, in currently recommended testing procedures there exists true risk for missing germline PVs in Lynch syndrome, as well as other conditions that may predispose patients to hereditary cancers and have characteristics overlapping the hallmarks of Lynch syndrome. The outcome can be misdiagnosis and undertreatment of patients. Second, IHC results are not reliable as supportive evidence for variant classification. Our findings support revisiting guideline recommendations for diagnostic testing of individuals diagnosed with CRC or other Lynch syndrome-related cancers with consideration given to first-line use of comprehensive germline panel testing that combines analytical accuracy with robust variant classification. This recommendation is aligned with the recent update of the NCCN guideline to consider germline multigene panel testing for all individuals with CRC (3).

## Data availability statement

The data analyzed in this study is subject to the following licenses/restrictions: Data are not publicly available due to patient privacy concerns, but can be provided upon reasonable request from the corresponding author. Requests to access these datasets should be directed to Shujuan Pan, span@myriad.com.

## Ethics statement

Ethical review and approval was not required for the study of human participants in accordance with the local legislation and institutional requirements. Written informed consent from the patients/participants OR patients/participants legal guardian/next of kin was not required to participate in this study in accordance with the national legislation and the institutional requirements.

## Author contributions

SP, JW, EM, DM-D, and BC contributed to conceptualization. HC contributed to data curation. HC contributed to formal analysis. SP contributed to methodology and project administration. HG, ML, KB, BR and DMD contributed to supervision. KB contributed to validation. SP wrote the original draft, and JW, KB and DM-D contributed additional writing, review, and editing. All authors contributed to the article and approved the submitted version.
