# Supplementary material for: Discordance between germline genetic findings and abnormal tumor immunohistochemistry staining of mismatch repair proteins in individuals with suspected Lynch syndrome
Source: Front Oncol. 2023 Jan 30;13:1069467. doi: 10.3389/fonc.2023.1069467 (PMC9923021; doi:10.3389/fonc.2023.1069467)
Supplement: Supplementary file 1 [file DataSheet_1.pdf]

Supplemental Table 1. Concordance between IHC patterns and germline MMR gene PVs. Green-shaded cells represent germline PVs expected by the corresponding IHC result. Red-shaded cells represent germline PVs that were unexpected based on the corresponding IHC result.

	**Germline PV**			
**IHC pattern**	***MLH1***	***MSH2/EPCAM***	***MSH6***	***PMS2***
MLH1				
MLH1/MSH2				
MLH1/MSH2/MSH6				
MLH1/MSH2/PMS2				
MLH1/MSH2/MSH6/PMS2				
MLH1/MSH6				
MLH1/MSH6/PMS2				
MLH1/PMS2				
MSH2				
MSH2/MSH6				
MSH2/MSH6/PMS2				
MSH2/PMS2				
MSH6				
MSH6/PMS2				
PMS2				

Supplemental Table 2. IHC patterns in cases with monoallelic *MUTYH* PVs identified.

**IHC patterns**	**Germline monoallelic *MUTYH* PV**
MSH6	c.1227_1228dupGG (p.Glu410Glyfs*43)
MSH2; MSH6	c.933+3A>C
MLH1; PMS2	c.934-2A>G
MLH1; PMS2	c.536A>G (p.Tyr179Cys)
MLH1	c.1187G>A (p.Gly396Asp)
MLH1	c.1187G>A (p.Gly396Asp)
